# Families of patients in ICU: A Scoping review of their needs and satisfaction with care

**DOI:** 10.1002/nop2.287

**Published:** 2019-05-18

**Authors:** Pamela Scott, Patricia Thomson, Ashley Shepherd

**Affiliations:** ^1^ Intensive Care Unit Forth Valley Royal Hospital Larbert UK; ^2^ School of Health Sciences University of Stirling Stirling UK

**Keywords:** anxiety and uncertainty, Family, intensive care, interventions, needs, satisfaction

## Abstract

**Aim:**

To describe published literature on the needs and experiences of family members of adults admitted to intensive care and interventions to improve family satisfaction and psychological well‐being and health.

**Design:**

Scoping review.

**Methods:**

Several selective databases were searched. English‐language articles were retrieved, and data extracted on study design, sample size, sample characteristics and outcomes measured.

**Results:**

From 469 references, 43 studies were identified for inclusion. Four key themes were identified: (a) Different perspectives on meeting family needs; (b) Family satisfaction with care in intensive care; (c) Factors having an impact on family health and well‐being and their capacity to cope; and (d) Psychosocial interventions. Unmet informational and assurance needs have an impact on family satisfaction and mental health. Structured written and oral information shows some effect in improving satisfaction and reducing psychological burden. Future research might include family in the design of interventions, provide details of the implementation process and have clearly identified outcomes.

## 1 INTRODUCTION

In the UK, 191,016 patients were admitted to the intensive care unit (ICU) in 2016. This figure rose to 193,813 in 2017 (Scottish Intensive Care Society Audit Group, (SIGSAG) (2017, 2018), Intensive Care National Audit and Research Centre, (ICNARC) 2017). With increases in the number of patient admissions to ICU come increases in poorer patient outcomes, for example, 20% of patients die prior to the hospital discharge or undergo a prolonged period of recovery (SIGSAG & ICNARC, 2017, 2017).

Admission to the ICU is often, although not always, unexpected, and the patient's condition is usually unstable (Delva, Vanoost, Bijttebeir, Lauwers, & Wilmer, 2002). Many ICU patients are unable to communicate with healthcare staff or participate in decision‐making about their treatment due to the severity of their illness, delirium or sedation (Mitchell, Burmeister, & E & Foster M, 2009). Consequently, healthcare professionals are increasingly approaching family members to speak for them and expanding the care and support provided from the patient to their family as well (Al‐Mustair, Plummer, O'Brien, & Clerehan, 2013). Involving the patient's family in the ICU stage of care is essential to enable healthcare providers to fully deliver person‐centred care. Often family members who know the patient best are not considered as part of the care team (Paul & Finney, 2015).

Admission to ICU, whether planned or unplanned, however means that family members may suddenly be faced with decision‐making and uncertainty about their relatives’ acute condition and prognosis (Paul & Rattray, 2008). Research suggests they are frequently overwhelmed by feelings of anxiety and worry due to fear of losing their loved one, deterioration of the family structure, concerns about the future, coupled with the stressful technological ICU environment (Bijttebeir, Vanoost, Delva, Ferdinande, & Frans, 2001; Delva et al., 2002). Up to 50% of relatives experience emotional distress or anxiety for up to two years after hospital discharge which influences their quality of life and lifestyle (Paul & Rattray, 2008). For these reasons, ICU care and quality measurement should include the families’ perspective of whether their needs were met or not, satisfaction with the care process and outcome and evaluation of interventions to improve their psychological health and well‐being (Flaatten, 2012). Current literature primarily focuses on healthcare professionals’ knowledge and understanding of family needs. It provides little insight from the perspective of the family as to what their experiences are, how they perceive the care delivered and the impact of having a loved one in ICU. There is limited research describing family experiences whilst in ICU and structured interventions that might support them during the patient's critical illness. The aim of this scoping review is to describe published literature on the needs and experiences of family members of adults admitted to intensive care and interventions to improve family satisfaction and psychological well‐being and health.

## 2 METHOD

The method adopted for this review was informed by Arskey and O'Malley (2005) scoping review framework. Scoping reviews are undertaken to examine the extent and nature of research activity in a particular field, to summarize and disseminate research findings and identify gaps in the literature (Arksey & O'Malley, 2005). The suggested steps in a scoping review are to: (a) identify the research questions; (b) identify relevant studies; (c) study selection; (d) chart the data; and (e) collate, summarize and report the results (Arksey & O'Malley, 2005). Scoping reviews do not address issues of quality appraisal but rather they have the potential to produce a large number of studies with different study designs and methodologies.

### 2.1 Research questions

The research questions posed before the literature search started were as follows:

1. What is currently known about family needs and family satisfaction with care?
2. What were the psychological symptoms experienced by family members in the ICU and the interventions available aimed at reducing those symptoms?

### 2.2 Identifying relevant studies and study selection

The search strategy involved searching the following electronic databases: Medline, Cinahl, Embase, Psycho Info, Science Direct and Cochrane library of systematic reviews and Google scholar. The search terms used included the following: family, intensive care, satisfaction, needs, interventions, anxiety and uncertainty. The search covered the period 1979–2017 as the first seminal study in this area was published in 1979. To be included in this review, published studies or prior literature reviews had to include relatives of adult critically ill patients admitted to the intensive care unit. Only published papers published or translated into English were included.

### 2.3 Charting the data

**Figure 1:**
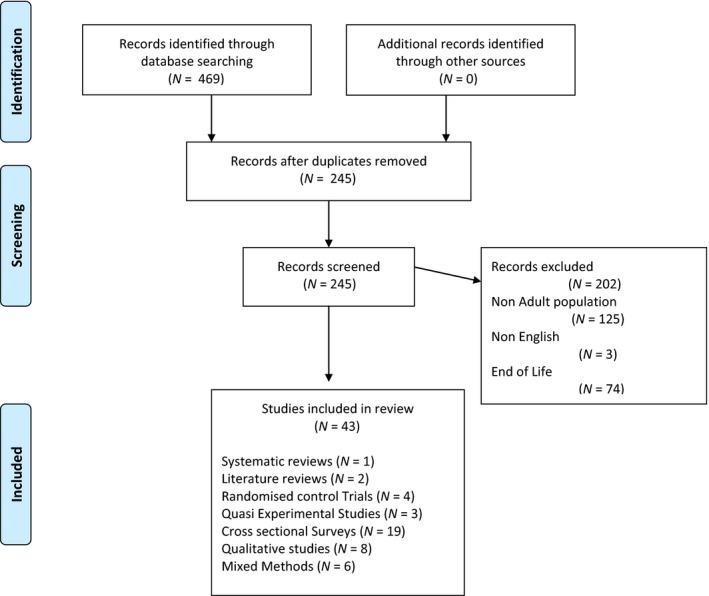
Article selection process for scoping review

The article selection process is summarized in Figure 1. Consistent with the approach proposed by Arksey and O'Malley, (2005), the findings from each paper selected were organized and key themes developed pertinent to the scoping aim.

A full list of articles were obtained and screened for duplicates by the lead author. Abstracts were examined to identify publications that met the inclusion criteria for this scoping review and reviewed by lead author. Reference lists of relevant articles and eligible primary research studies or reviews were checked by hand to identify articles not captured by electronic searches.

### 2.4 Collating, summarizing and reporting results

To enable a logical and descriptive summary of the results, data were extracted using the following key headings: authors(s), year of publication and title of publication; country of origin; study design; sample size; sample characteristics; intervention type and outcome.

### 2.5 Ethics

Research Ethics Committee approval was deemed not required as this was a scoping review.

## 3 RESULTS

In total, 468 published papers were retrieved. Removing duplicates and screening abstracts and full texts resulted in the inclusion of 43 published articles which included 40 research studies, one systematic review and two literature review (Figure 1). The quantitative research studies included four randomized control trials, three quasi‐experimental studies and 19 cross‐sectional surveys. The qualitative research included two grounded theory studies and six other studies that employed a qualitative approach although no specific design was specified. A further six studies used a combination of quantitative and qualitative approaches. The papers retrieved were published in journals aimed at the medical profession (*N* = 21), followed by nursing (*N* = 20), psychology (*N* = 1) and social work (*N* = 1). Most of the studies were conducted in the USA (*N* = 13), followed by Canada (*N* = 4), France (*N* = 4), Denmark/Norway/Sweden (*N* = 4), Hong Kong (*N* = 3), Australia (*N* = 3), Belgium (*N* = 3), Jordan/Iran (*N* = 2), UK (*N* = 3), Germany (*N* = 1), Greece (*N* = 1), Turkey (*N* = 1) and Spain (*N* = 1). The settings were specified as general ICUs, which incorporated medical, surgical, neurological and trauma patients (*N* = 35) and neurological ICU (*N* = 5).

Four key themes were identified from the scoping review: (a) Different perspectives on meeting family need; (b) Family satisfaction with care in ICU (c) Factors having an impact on family well‐being and their capacity to cope; and (d) Psychosocial interventions**.**

### 3.1 Theme 1 different perspectives on meeting family need

Under Theme 1, two key areas related to meeting family needs were identified, namely family member's perceptions of their needs and the healthcare team's perceptions of family needs.

#### 3.1.1 Family member's perception of their needs

Four quantitative studies (Auerbach et al., 2005; Lee & Lau., 2003; Molter, 1979; Omari, 2009) and three qualitative studies (Bond, Draeger, Mandleco, & Donnelly, 2003; Fry & Warren, 2007; Keenan & Joseph, 2010) were identified, and one literature review (Verhaeghe, Defloor, Zuuren, Duijnstee, & Grypdonck, 2005) explored family members’ perceptions of their needs (Table 1). All four quantitative studies used the Critical Care Family Needs Inventory (CCFNI), a 45‐item self‐report questionnaire that assessed family needs within five dimensions: support, comfort, information, proximity and assurance (Molter, 1979). Most studies were single centre. Family needs data were obtained during the acute phase of critical illness (first 24–72 hr). The most important family needs identified were for information and assurance, followed by proximity, comfort and support, respectively. A recent literature review concluded that information and assurance appeared to be the greatest universal needs of family members of critically ill patients (Al Mustair et al., 2013; Verhaeghe et al., 2005). Families want timely, clear and understandable information about their relative's medical condition, but without leaving room for unrealistic hope.

**Table 1:** Studies of family needs

Author	Aim	Setting	Sample size	Method	Outcome
Auerbach et al. (2005)	To examine family members perceptions of whether their needs were met in a trauma ICU at both at admission and prior to discharge	One Trauma ICU in teaching hospital in the United States (USA)	Forty family members	Quantitative CCFNI	On Admission,most prominent of unmet needs were information, explanations and comfortable waiting area Discharge—tended to show all needs were being met
Bijttebier et al. (2001)	To investigate differences between perceptions of family members, physicians and nurses about the needs of relatives of critical care patients.	One general ICU of a University Hospital in Belgium	Two hundred family members, 38 physicians, 143 nurses	Quantitative CCFNI	Information emerged as being the most important factor across all three groups. Nurses and physicians underestimated this need.
Bond et al. (2003)	To describe the needs of families of patients with severe traumatic brain injury in a neurosurgical ICU	One neurological ICU in trauma centre USA	Seven family members	Qualitative‐Exploratory interviews	Content analysis of the interviews identified 4 themes The need to know, The need for consistent information, The need for involvement The need to make sense of the experience
Fry and Warren (2007)	To describe the perceived needs of the ICU family members viewed from their own words	One General ICU in the USA	Fifteen family members	Qualitative‐Contextual analysis using interviews	4 explicit needs were expressed by all participants. These needs were seeking information. Trusting the professionals. Being a part of the care and maintaining a positive outlook.
Hinkle et al. (2009)	To describe family members needs of ICU patients identified by family members and nurses.	Six ICU's (4 neurological and 2 surgical) in the USA	Hundred and one family members and nurses	Qualitative‐descriptive approach	Hierarchical cluster analysis identified the 4 themes of Emotional resources and support Trust and facilitation of needs Treatment information Feelings Family members and nurses differed significantly on three of the four themes
Hinkle & Fitzgerald (2011)	Needs of American relatives of intensive care patients: Perceptions of nurses, physicians and relatives	Six ICU's (4 neurological and 2 surgical) in the USA	Hundred and one family members, 28 physicians and109 nurses	Quantitative CCFNI	The three most important needs were 1)To have questions answered honestly 2)To be assured that the best care possible is being given to the patient 3)To feel the hospital personnel care about the patient.
Keenan and Joseph (2010)	Identify the needs of family members of ICU patients who have sustained a severe traumatic brain injury	One neurological ICU in Canada	Twenty‐five family members	Qualitative Semi‐structured Interviews	Key themes identified were as follows: The need to talk about their experience. To receive information about the injury and prognosis. To be supported by professionals in becoming involved in their relative's care.
Kinrade et al. (2010)	To investigate the needs of relatives whose family member is unexpectedly admitted to the ICU and compare them with nurses perspectives of family needs	One general ICU in Australia	Twenty‐five family members, 33 nurses	Quantitative CCFNI	The importance of the need for information provision and communication between family members and ICU staff was identified of key importance
Lee and Lau (2003)	To identify the immediate needs of family members in a general ICU	One general medical, surgical and neurological ICU in Hong Kong	Forty family members	Quantitative CCFNI	Reassurance and Proximity‐most important unmet needs
Leung et al. (2000)	To identify family members perceptions of immediate needs within 48–96 hr following admission of a relative to critical care	One general ICU in Hong Kong	Thirty‐seven family members, 45 registered nurses	Quantitative CCNFI	Top need for families was assurance and for nurses it was information.
Molter (1979)	To identify the needs of relatives of critically ill patients.	Two general ICU in the USA	Forty family members	Quantitative CCNFI	Top three needs were as follows: Assurance, Information and proximity
Omari 2009	To identified the perceived needs of family members who have a family member admitted to the ICU	Six general ICUs in 3 hospitals in Jordan: Ministry of Health, university hospital, and private hospital	Hundred and thirty‐nine family members	Quantitative CCFNI	The Assurance and Information subscales were perceived as the most important, but the needs associated with these items were met inconsistently
Ozbayir et al., 2014	To compare intensive care nurses and relatives perceptions about intensive care family's needs	A general ICU in one teaching hospital in Turkey	Seventy family members, 70 registered nurses	Quantitative CCFNI	The CCFNI rankings for the two groups were similar for eight out of the ten most highly ranked items but differed in order. Families ranked assurance and information as key priorities. Nurses ranked proximity, assurance then information
Takman and Severinsson (2006)	To describe and explore nurses and physicians perceptions of relatives needs	Eight medical and surgical ICUs in Norway and Sweden	Ninety‐seven Registered Nurses and 5 Physicians	Quantitative and Qualitative CCFNI plus 1 open‐ended item	Qualitative content analysis —Identified four categories: ‐The need to feel trust in the healthcare providers’ ability ‐The need for ICU and other hospital resources, ‐The need to be prepared for the consequences of critical illness and “patients” needs ‐Reactions in relation to significant others

There was generally consistency across studies in how the importance of these needs is ranked, although some variations do occur (Auerbach et al., 2005; Lee & Lau, 2003), which were attributed to differences in patient's severity of illness, cultural expectations, differences in ICU practices and healthcare systems (Lee & Lau, 2003; Verhaeghe et al., 2005). Age, gender, relationship to the patient, length of patient stay in the ICU and patient diagnosis were not found to be correlated with family members' ranking of needs (Omari, 2009; Verhaeghe et al., 2005).

The qualitative studies of family member's perceptions of need provide a deeper understanding of family needs whilst in the ICU. All qualitative data describe that family members feel the need to create an alliance with healthcare staff and that this had a positive impact on their ability to handle the situation they are being faced with (Bond et al., 2003; Fry & Warren, 2007; Keenan & Joseph, 2010). Families who were confident and trusting in healthcare staff's ability to care for their relative felt more able to leave at night and take care of both themselves and their other family members (Fry & Warren, 2007). Those who perceived a lack of trust or engagement with healthcare staff describe difficulty in coping, lack of confidence, hesitancy to ask questions and dissatisfaction with care provided (Fry & Warren, 2007). Bond et al. (2003) described that inclusion of family members by the ICU team not only increased their understanding of the gravity of the patient's situation but helped prepare them for their potential caregivers role on discharge from hospital.

#### 3.1.2 Healthcare teams perceptions of family needs

Few studies have evaluated the ability of healthcare staff to meet and satisfy the needs of ICU family members. Three single‐centre quantitative studies (Kinrade, Jackson, & Tomany, 2010; Leung, Chien, & Mackenzie, 2000; Ozbayir, Tasdemir, & Ozseker, 2014) and one multicentre qualitative study included only nursing staff (Hinkle, Fitzpatrick, & Oskrochi, 2009) (Table 1). Three studies, two of which were multicentre, evaluated both medical and nursing staff perspectives of family needs, two using quantitative methods (Bijttebier et al., 2001; Hinkle & Fitzgerald, 2011) and one mixed methods (Takman & Severinsson, 2006). Healthcare staff ranked the need for information and assurance as the top two important needs in all studies. Yet, despite this, both needs were the most frequently cited by family members as being unmet by healthcare staff (Hinkle et al., 2009; Leung et al., 2000; Omari, 2009). Unmet needs were reported to occur because ICU nurses and doctors do not perceive family needs accurately, undervalue their role and/or fail to sufficiently support the family (Bijttebeir et al., 2001; Hinkle et al., 2009; Leung et al., 2000). The patient's illness severity may also mean that the time available for communication with healthcare staff is limited and the ability to engage in discussion is compromised by the patient's clinical condition (Bijttebeir et al., 2001). Interestingly, age, gender, academic qualifications and working experience did not predict the healthcare providers’ ranking of needs of the family of the critically ill patient (Takman & Severinsson, 2006).

### 3.2 Theme 2 Family satisfaction with care in ICU

Seven studies, four of which were large multicentre studies, investigated family satisfaction with care and decision‐making in the ICU. Three studies used quantitative methods (Heyland, Rocker, & Dodek, 2002; Hunziker et al., 2012; Hwang et al., 2014) and four were mixed methods studies (Clark, Milner, Beck, & Mason, 2016; Hendrich et al., 2011; Karlsson, Tisell, Engrstom, & Andershed, 2011; Schwarzkopf et al., 2013). No qualitative studies of family satisfaction with care in ICU were found (Table 2). Six of the quantitative studies evaluated family satisfaction using the Family Satisfaction‐ICU (FS‐ICU) questionnaire and one used the Critical Care Family Satisfaction Survey (CCFSS).

**Table 2:** Family satisfaction studies

Author	Aim	Setting	Sample Size	Method	Outcome
Clark et al. (2016)	To measure family satisfaction with care in a medical and surgical ICU	One general ICU in America	Forty family members	Quantitative/Qualitative FS‐ICU with analysis of qualitative questions	Overall, family satisfaction with care and decision‐making was good. 50% of family members reported the need for more timely and accurate information
Hwang et al. (2014)	To describe family satisfaction with care in a Neurological ICU and Medical ICU	One neurological ICU in America	Hundred and twenty‐four family members	Quantitative FS‐ICU	Less than 60% of ICU's families were satisfied by frequency of physician communication
Heyland et al. (2002)	To determine the level of satisfaction of family members with the care that they and their critically ill relative received	Six general ICUs at university hospital across Canada	Six hundred and twenty‐four family member	Quantitative FS‐ICU	Majority of respondents satisfied with overall care and decision‐making. Greatest satisfaction with nursing skill and competence, compassion and respect and pain management. Least satisfied with frequency of communication and waiting room atmosphere
Hendrich et al., 2011)	To describe the qualitative findings from a family satisfaction survey	Twenty three mixed ICUs across Canada	Eight hundred and eighty‐eight family members	Qualitative/Quantitative FS‐ICU with analysis of qualitative questions	Six themes identified central to family satisfaction. Positive comments were more common for: quality of the staff (66% vs. 23%), overall quality of medical care provided (33% vs. 2%), and compassion and respect shown to the patient and family (29% vs. 12%). Positive comments were less common for: communication with doctors (18% vs. 20%), waiting room (1% vs. 8%), and patient rooms (0.4% vs. 5%)
Hunziker et al. (2012)	To determine what factors ascertainable at ICU admission predicted family members dissatisfaction with ICU care	Nine mixed ICUs in the USA	Four hundred and forty‐five family members	Quantitative FS‐ICU	The most strongly associated factors reported by families relate to nursing competence, followed by completeness of information, and concern and caring of patients by intensive care unit staff
Karlsson et al. (2011)	To describe family members satisfaction with the care provided in a Swedish ICU	One general ICU in Sweden	Thirty‐five family members	Quantitative/ Qualitative Critical Care Family Satisfaction Survey (CCFSS)	Family members need for regular information was highlighted. The ICU staff's competence was also seen to be important for family members satisfaction with care
Schwarzkopf et al. (2013)	To assess family satisfaction in the ICU and areas for improvement using quantitative and qualitative analyses	Four (2 surgical, 1 medical and 1 neurological) ICUs in a hospital in Germany	Two hundred and fifty family members	Qualitative/Quantitative FS‐ICU with analysis of qualitative questions	Overall satisfaction with care and satisfaction with information and decision‐making based on summary scores was high. No patient or family factors predicted overall satisfaction, including patient survival

Research study findings suggest that families of the critically ill are highly satisfied with the care their relative receives, especially with aspects of care about skill and competence of staff and the respect given to the patient (Clark et al., 2016; Hendrich et al., 2011; Heyland et al., 2002; Hunziker et al., 2012; Hwang et al., 2014; Schwarzkopf et al., 2013). Families were less satisfied with emotional support, the provision of understandable, consistent information and coordination of care (Clark et al., 2016; Hendrich et al., 2011; Heyland et al., 2002; Hunziker et al., 2012; Hwang et al., 2014; Schwarzkopf et al., 2013). Families felt more satisfied when clear, honest information was delivered to them in understandable language as this enables them to actively participate in the decision‐making process (Heyland et al., 2002; Hunziker et al., 2012; Hwang et al., 2014). One study by Heyland et al. (2002) found completeness of information was the single most important factor accounting for the variability in overall satisfaction. Families who rated the completeness of information highly were much more likely to be completely satisfied with their ICU experience. In another study, families were less satisfied not by the delivery of information received but by the lack of information received from medical staff (Hwang et al., 2014). When family satisfaction with care was measured using the CCFSS, overall satisfaction with care was high, however, similar to Hwang et al., (2014), dissatisfaction among some family members related to the lack of availability of medical staff for regular meetings (Karlsson et al., 2011).

Reporting on the three open‐ended questions in the FS‐ICU, three of the six studies provided further knowledge of family member's experiences with care delivery in the ICU (Clark et al., 2016; Hendrich et al., 2011; Schwarzkopf et al., 2013). In the free‐text responses, families expressed the need for better communication with healthcare staff and the need for timely, accurate and up‐to‐date information about changes in their relative's condition.

### 3.3 Theme 3 Factors having an impact on family well‐being and capacity to cope

Two key factors were identified in relation to the factors impacting on family well‐being and capacity to cope, namely anxiety and uncertainty.

#### 3.3.1 Anxiety

Eight studies examined anxiety in family members of the critically ill (Table 3). Seven of these studies adopted quantitative approaches (Day, Bakin, Lubchansky, & Mehta, 2013; Delva et al., 2002; Paparringopoulos et al., 2006; Pochard et al., 2001, 2005; Rodriguez & San Gregorio, 2005; Young et al., 2005) and one study a qualitative approach (Iverson et al., 2014). Most studies were single centre. Levels of anxiety in family members were mainly measured 24–72 hr after the patient's admission to ICU. The prevalence of anxiety symptoms in these studies ranged from 40%–73% (Pochard et al., 2005). Risk factors associated with an increase in symptoms of anxiety included being female, a spouse, an unplanned ICU admission, lower educational status, poor sleep pattern, fatigue, lack of regular meetings with medical staff and failing to meet family needs (Day et al., 2013; Delva et al., 2002; Paparringopolous et al., 2006; Pochard et al., 2001, 2005). Whilst symptoms may reduce over time, Paul and Rattray, (2008) in a recent review of the literature highlighted that moderate to high levels of anxiety are present for up to 2 years after hospital discharge in relatives providing care after ICU.

**Table 3:** Studies of psychological outcomes

Author	Aim	Setting	Sample Size	Method/Measures	Outcome
Agard and Harder (2007)	To explore and describe the experiences of relatives of critically ill adults	One neurosurgical and One General ICU in Denmark	Four spouses and 3 parents	Qualitative Grounded theory	Relatives were both vulnerable and resourceful simultaneously. They tried to fit in though using 3 strategies Enduring uncertainty Putting self aside Forming personal cues They needed information all of the time and if not received formed their own personal cues leading to misunderstandings
Burr (1998)	To explore family needs and experiences and gain insight into nurse/family roles	Mixed ICU in teaching four hospitals in Australia	Hundred and five family members CCFNI 26 Interviews	Quantitative/Qualitative CCFNI/ semi‐structured interviews	Two major needs emerged from the interviews that are not represented on the CCFNI: The need of family members to provide reassurance and support to the patient; and their need to protect
Day et al. (2013)	To investigate sleep quality, levels of fatigue and anxiety in families of critically ill adults	One medical and surgical ICU in Canada	Ninety‐four family members	Quantitative General Sleep disturbance scale Beck Anxiety Inventory Scale Lee's Numerical Scale for fatigue	The most common factor associated with poor sleep was anxiety (43.6%), tension (28.7%) and fear (24.5%). The need for more information and greater frequency of updates was cited by family members as a possible solution for reducing anxiety and promoting sleep
Delva et al. (2002)	To explore the needs and anxiety of family members of patients admitted to the ICU	One surgical ICU and One medical ICU in Belgium	Two hundred family members	Quantitative State‐Trait Anxiety Inventory (STAI) CCFNI	The younger the patient the more anxious the family member was (*p*=0.0048). Females were more anxious than males (*p* < 0.01) and state anxiety was higher with non‐planned rather than planned admissions (*p*<0.01).Lower educational level predicted higher anxiety (*p*<0.001) Top 2 needs identified were for information and assurance
Iverson et al. (2014)	To explore surrogate decision makers challenges	Two general ICUs in the USA	Thirty‐four family members	Qualitative Semi‐structured Interviews	Anxiety influenced surrogate decision makers confidence in making decisions. This stress can be minimized by improving communication between these family members and the medical team
Jamerson et al. (1996)	To describe the experiences of families with a relative in ICU	One surgical/trauma ICU in the USA	Twenty family members	Qualitative Focus Groups	4 categories of experiences were identified: Hovering is an initial sense of confusion and uncertainty, Information seeking is a tactic used to move out the hovering stage and to identify the patients’ progress Tracking is the process of observing, analysing and evaluating patient care Garnering of resources is the act of acquiring what the family members perceive as needed for themselves or their relative. Families experience a sense of uncertainty resolved by seeking information and resources
Johansson et al. (2005)	To gain an understanding of what relatives experience as supportive when faced with the situation of having a next of kin admitted to ICU	One general ICU in Sweden	Twenty‐nine family members	Qualitative Grounded theory	The ICU situation for relatives was characterized by uncertainty as to whether the patient would survive or suffer functional impairment, and a fear of complications arising
Pochard et al. (2001)	To determine the prevalence and factors associated with symptoms of anxiety and depression in family members of ICU patients	Fort‐three mixed (37 adult and six paediatric) ICUs in France	Nine hundred and twenty family members	Quantitative HADS	Symptoms of anxiety and depression common (69.1% and 35.4%, respectively) among family members visiting patients 3–5 days after admission to the ICU. Symptoms of anxiety were independently associated with being the spouse, female, lack of regular meetings with nursing and medical staff symptoms of depression were also associated being the spouse, female sex, contradictions in information
Pochard et al. (2005)	To determine the prevalence and factors associated with symptoms of anxiety and depression in family members at the end of ICU stay	Seventy‐eight mixed ICUs in France	Five hundred and forty‐four family members	Quantitative Hospital Anxiety and Depression Scale (HADS)	Symptoms of anxiety and depression common (73.4% and 35.3%, respectively) at the end of their ICU stay. Symptoms of depression were more prevalent in non‐survivors (48.2%) than survivors (32.7%). A high severity of illness and younger patient age on admission predicted both anxiety and depression
Paparrigopoulos et al. (2006)	To evaluate the short term psychological impact on family members of intensive care patients during their stay in ICU	Two general ICUs in Greece	Thirty‐two family members	Quantitative: Centre for Epidemiological Depression scale, the State‐Trait Anxiety Inventory (STAI) and the impact event scale.	Symptoms of anxiety, depression and post‐traumatic stress common (60.4%, 97% and 81%, respectively) at first assessment. On second assessment, symptoms decreased but remained high (47%, 87% and 59%). Females and spouses exhibited higher levels of anxiety
Rodriguez &San Gregorio (2005)	To evaluate whether certain variables (Anxiety, depression, Quality of life) impacted on family members on ICU admission and 4 years later	One Neurosurgical ICU in Spain	Fifty‐seven family members	Quantitative Psychosocial questionnaire developed by authors Clinical Analysis Questionnaire Family Environment Scale Fear of Death Scale	High anxiety depression, apathy withdrawal and paranoia scores were high during ICU admission compared to scores obtained 4 years later Relative's scores for “fear of their own death” were lower on ICU admission compared to 4 years later
Young et al. (2005)	To investigate symptoms of anxiety and depression in patients and families after ICU discharge	ICU follow‐up clinic in the UK	Fifteen family members, 20 relatives	Quantitative HADS	Relatives were more anxious than patients

#### 3.3.2 Uncertainty

Five qualitative mainly single‐centre studies explored the uncertainty that families face when a relative is admitted to ICU and how this contributes to feelings of anxiety and inability to cope with the magnitude of the situation (Agard & Harder, 2007; Burr 1998; Iverson et al., 2014; Jamerson et al., 1996; Johansson, Hildingh, & Fridlund, 2005) (Table 3). Families describe their ongoing uncertainty about whether their family member will survive or suffer permanent disability, and having the daily fear of complications arising (Johansson et al., 2005). The need to seek out information on the patient's condition and prognosis was a consistent theme in all the studies. Families felt they should always be at the bedside; they searched for cues from healthcare staff that indicated an improvement or deterioration in the patient's condition (Agard & Harder, 2007; Burr, 1998). When these cues were absent, symptoms of anxiety manifest due to the uncertainty of the situation and they sought reassurance from staff that their relative was in safe hands. It was the “not knowing” that was the worst part of their entire ICU experience which often lead to misunderstandings and profound feelings of uncertainty, anxiety and distress until enough information was given or obtained (Agard & Harder, 2007; Burr, 1998; Iverson et al., 2014). In one study, Iverson et al. (2014) reported the role of surrogate decision maker amplified family members’ anxiety at an already challenging time; they were afraid that they were making the “wrong” decision on behalf of their loved one.

### 3.4 Theme 4 Psychosocial interventions

Seven studies investigated interventions to improve family needs, family satisfaction with care and anxiety and depression. These studies included four randomized controlled trials (RCT's) (Azoulay et al., 2002; Jones et al., 2004; Lautrette et al., 2007; Yousefi, Karami, Moeini, & Ganji, 2012) (Table 4) and three quasi‐experimental studies (Appleyard et al., 2000; Chien, Chui, Lam, & LP WY., 2006; Mitchell et al., 2009) (Table 4). Two of the RCTs examined family satisfaction with care as the primary outcome (Azoulay et al., 2002; Yousefi et al., 2012), whilst two trials investigated post‐traumatic stress disorder (PTSD) and symptoms of anxiety and depression as outcomes (Jones et al., 2004; Lautrette et al., 2007). Two quasi‐experimental studies investigated the effect of needs‐based interventions on family satisfaction (Appleyard et al., 2000; Chien et al., 2006), and a third study examined respect, collaboration and support (Mitchell et al., 2009).

**Table 4:** Psychosocial Interventions

Author	Aim	Setting	Sample Size	Method/Measures	Outcome
Appleyard et al. (2000)	To gain knowledge and understanding of the role of volunteers pay in the critical care family waiting room	One general ICU in the USA	Fifty‐eight family members	Quantitative Quasi‐experimental study with pre and post‐test design, without control group	Increased family satisfaction from comfort needs only
Azoulay et al. (2002)	To determine whether a standardized family information leaflet improved satisfaction and comprehension of the information provided to family members of ICU patients	Thirty‐four General ICU in France	Family members Intervention Group = 87 Control Group = 88	Quantitative A multicentre, prospective, randomized controlled trial (RCT) (Blinded)	Increased family satisfaction and improved comprehension of information
Jones et al. (2004)	To evaluate the effectiveness of the provision of information in the form of a rehabilitation programme following critical illness in reducing psychological distress in the patients’ close family.	Three General ICU in UK	Family members Intervention Group = 56 Control Group = 46	Quantitative Randomized controlled trial, blind at follow‐up with final assessment at 6 months.	High incidence of psychological distress which did not reduce postintervention
Chein et al. (2006)	To examine the effect of a needs‐based education programme provided within the first 3 days of patients' hospitalization, on the anxiety levels and satisfaction of psychosocial needs of their families.	One General ICU in Hong Kong	Family members Intervention group = 34. Control Group = 32	Quantitative Quasi‐experimental with pre‐ and post‐test design.	Reduced anxiety. Increased satisfaction of family members.
Lautrette et al. (2007)	To evaluate the effect of a proactive communication strategy that consisted of an end‐of‐life family conference conducted according to specific guidelines and that concluded with the provision of a brochure on bereavement.	Twenty‐two (10 medical, 3 Surgical and 9 General) ICUs in France	Family members Intervention Group = 56 Control group = 52	Quantitative Multicentre RCT	Decreased the risk of symptoms of post‐traumatic stress disorder, anxiety and depression
Mitchell et al. (2009)	To evaluate the effects on family‐centred care of having critical care nurses partner with patients’ families to provide fundamental care to patients.	Two General ICUs in the USA	Family members Intervention Group = 99 Control Group = 75	Quantitative Quasi‐experimental with pre‐ and post‐test design	Improved respect, collaboration, support and overall scores of family‐centred care
Yousefi et al. (2012)	To determine the effectiveness of nursing interventions based on family needs on family satisfaction level of hospitalized patients in the neurosurgery ICU	One neurosurgical ICU in Iran	Family members Intervention group = 32, Control group = 32	Quantitative Multicentre RCT	Increased satisfaction of families

Overall, a diverse range of interventions were used in these studies with the aim of improving the number of family needs met, improving satisfaction and psychological well‐being. Azoulay et al. (2002) distributed a family information leaflet to supplement standardized family meetings to assess whether it improved their understanding of diagnosis and proposed interventions. The leaflet improved comprehension of diagnosis and treatment but not of prognosis. The authors attributed this to the focus of the leaflet being on diagnosis and treatment and that understanding the prognosis is difficult for families. Satisfaction with care did not significantly differ between the two groups. However, although not statistically significant they reported the family information leaflet did improve satisfaction among those family members with good comprehension. Yousefi et al. (2012) examined whether family satisfaction was improved by allocating families with a dedicated ICU support nurse. The intervention was based on “family needs inventory” where the ICU nurses role was to provide accurate explanations and information to families about the patient and their critical illness. Information and explanations were given about the ICU environment, equipment and personnel as well as treatment, diagnosis and prognosis. Meetings with the physician and allied health professionals were also facilitated. Satisfaction in the intervention group was significantly increased postintervention. Lautrette et al. (2007) introduced use of a bereavement brochure along with a proactive family conference for relatives of patients in ICU with high likelihood of mortality. They found significantly fewer symptoms of post‐traumatic stress disorder (PTSD), anxiety and depression after 90 days. In contrast, Jones et al. (2004) failed to show the provision of general written information around recovery after ICU delivered by nurses in 3 ICUs reduced anxiety, depression and PTSD symptoms at eight weeks and six months after ICU discharge. Some relatives remained anxious, and they met criteria for PTSD.

Other studies have looked at the effect of relatives assisting with the provision of care to the patient (Appleyard et al., 2000; Chien et al., 2006; Mitchell et al., 2009). Results from quasi‐experimental studies suggest better family satisfaction and reduced emotional distress postintervention, compared with the usual care group (Appleyard et al., 2000; Chien et al., 2006; Mitchell et al., 2009). For example, Chien et al. (2006) found that performing needs‐based training on the patient's family needs assessed on admission to ICU, decreased anxiety and increased their satisfaction. The intervention itself was labour intensive, and further research is required to identify which specific aspects of the programme were effective. Further, Appleyard et al. (2000) reported greater family satisfaction about comfort needs following the introduction of a volunteer programme in the ICU but no differences were found for the other CCFNI factors, including information, assurance, proximity and support. Notably, the volunteers reported the nurses became more communicative and more concerned about families’ needs following the introduction of the intervention. In the third study, Mitchell et al. (2009) reported that encouraging patient's family members to assist in providing care to their relatives significantly improved respect, collaboration, support and overall satisfaction. This study, however, only included the relatives of long‐term ICU patients with a length of stay greater than 11 days, thereby limiting the results to this group.

## 4 DISCUSSION

To the best of our knowledge, this is the first scoping review to describe published literature on the needs and experiences of family members of adult critically ill patients and interventions to improve family satisfaction and psychological health and well‐being. Forty research studies and three review articles were included in the review.

Family needs were investigated primarily through use of the CCFNI which highlights the most pressing family needs as being for information and reassurance followed by proximity, comfort and support, respectively. Families want honest and up‐to‐date information delivered daily in understandable terms about their relative's progress, without leaving room for unrealistic hope (Auerbach et al., 2005). They also want to be contacted anytime of the day or night if their relative's clinical condition changes and to be reassured they are receiving the best possible care (Omari, 2009). From their experiences, families felt there was a need to develop a trusting and mutually respectful relationship with healthcare staff and that this helped them adjust to the situation they were faced with (Bond et al., 2003; Fry & Warren, 2007; Keenan & Joseph, 2010).

Fulfilling family needs is important as unmet needs leave family members feeling uninformed, dissatisfied and disenfranchised from clinical decision‐making and with the day‐to‐day care of their relative (Wall, Curtis, Cooke, & Engelberg, 2007). The ability to meet or satisfy family needs is one of the main challenges that healthcare staff encounter in the ICU. Even if families’ needs are known to ICU staff, studies have indicated that these needs are not always met (Hinkle et al., 2009; Leung et al., 2000; Omari, 2009).

To improve the quality of care provided to families assessing families’ satisfaction with the patient care delivered, particularly in ICU, is important for several reasons. Firstly, healthcare providers need to develop open collaborative and supportive relationships with family members to enable them to cope with their distress and speak for the patient. Secondly, the collection of objective data on family satisfaction is desirable to assess how well healthcare providers are doing in this area. Data on family satisfaction are measured as a surrogate marker of the quality of their care (Heyland & Tranmer 2001).

Key areas for improvement identified were including the family as part of the ICU team, increasing open communication and assessing and potentially revisiting their level of understanding of the information they have been given (Clark et al., 2016; Hendrich et al., 2011; Heyland et al., 2002; Hunziker et al., 2012; Hwang et al., 2014; Schwarzkopf et al., 2013). Nurses who are in constant close contact with families are in an ideal position to ensure that family information and assurance needs are met. However, according to research, some nurses lack confidence in providing information, often being afraid of not giving the correct information or not providing adequate answers (Engstrom & Soderberg, 2007; Soderstrom, Saveman, Hagberg, & Benzein, 2003; Stayt, 2007). This is thought to be the case because nurses believe they are educationally underprepared and not sufficiently qualified to give the level of information required (Krimshtein et al., 2011; Stayt, 2007). Medical staff on the other hand have difficulty meeting with families and providing regular information delivered in a way families understand (Heyland et al., 2002; Hunziker et al., 2012; Hwang et al., 2014). Poor communication skills, insufficient training, delivering patient rather than family‐centred care and a lack of time have been attributed to this (Azoulay et al., 2000; Bijttebeir et al., 2001; Moreau et al., 2004).

Several studies highlighted additional factors that have an impact on family needs being met and their capacity to cope. Symptoms of anxiety are elevated at the onset of critical illness, and the uncertainty of their family members condition exacerbate these symptoms (Pochard et al., 2005). From clinical experience and research, high levels of anxiety and uncertainty result in family members overestimating or underestimating the risks and/or benefits of clinical treatments, impairs comprehension and decision‐making capabilities (Azoulay et al., 2000; Pochard et al., 2001). Anxiety therefore has important implications for family members who participate regularly in decisions about the care of their relative. Providing timely information, and preparing families for transitions in the delivery of care, may minimize the uncertainty and anxiety they experience (Azoulay et al., 2000).

Identifying interventions for supporting family members of the critically ill during the acute phase of their illness is necessary because if their relative survives, they are likely to care for them during a prolonged and often difficult recovery period (Pochard et al., 2005). The components of the interventions reviewed included a range of tools or strategies, for example family information booklet, bereavement brochure, structured meetings and dedicated nurse support (Appleyard et al., 2000; Azoulay et al., 2002; Chien et al., 2006; Jones et al., 2004; Lautrette et al., 2007; Mitchell et al., 2009; Yousefi et al., 2012).

From the intervention studies reviewed, providing a combination of targeted written and oral information delivered by nursing and medical staff caring for the patient significantly increased satisfaction and reduced anxiety with this reduction being sustained over time (Chien et al., 2006; Lautrette et al., 2007; Yousefi et al., 2012). Reasons for this pattern are because families were provided with good knowledge about their relative's clinical condition and treatment and contacted through the day either by phone or by attending a family meeting. These phone calls or meetings ensured families received updated information, had an opportunity to get questions answered and support when difficult decisions needed to be made. Additionally, families conveyed greater satisfaction with needs met if they received information about the ICU environment and equipment either through leaflets or discussions with staff and were involved in care of the patient at the bedside (Lautrette et al., 2007). Thus, not maintaining continuous and multiple methods of communication with the family delivered by the ICU team could account for the lack of positive statistically significant results in the other intervention studies (Appleyard et al., 2000; Azoulay et al., 2002; Jones et al., 2004; Mitchell et al., 2009).

Providing high‐quality information in a variety of ways ensuring that family members understand the nature of their relative's condition, including diagnosis, prognosis and treatment risks and benefits, is crucial for family members to cope with their role as substitute decision makers (Azoulay et al., 2000, 2001; Bond et al., 2003). Azoulay, Kentish‐Barnes, and Nelson (2016) suggest that discussions with families open with the question “What is your understanding of what the clinical team expects to happen?” or “What has the team told you about what to expect?” If the answer differs from that of the medical staff, then this is the best place to start to identify the source of the discordance. Intensive care units that are able to support interventions based on meeting family information needs, in addition to reducing psychological burden and increasing satisfaction, will enable each family to provide more support to their relative in the ICU.

### 4.1 Limitations of the review

Only English‐language articles were considered for inclusion in this scoping review. As such, this review misses potentially relevant articles written in other languages, which primarily covers research conducted in America. Most of the studies in this review involved female family members of the critically ill. Most studies obtained data from family members within 24–72 hr of admission to the ICU, which could affect the validity of the data because family members experience intense emotions and stress during these times.

Although experimental studies were identified, there were some methodological weaknesses. Most studies were descriptive, non‐experimental, single‐centre studies with small sample sizes, as such their findings may not be generalizable. There was an absence of theory to frame or guide the intervention, and each study identified limitations in their study design and outcome measures. Differences in study design, population, the number of samples and methods of intervention make it difficult to compare the results. Several of the studies measured the effect of the interventions in reducing family's anxiety; however, it is difficult to ascertain whether the reduction in anxiety is because of the intervention itself or the level of severity of the patient's illness.

### 4.2 Future research

There is a need for further empirical research to increase understanding of family needs and their perspective of whether their needs were met or not and the factors that militate against this. Differences in perceptions of need should be identified and examined from the perspectives of family and ICU staff over time. More studies are needed into the effectiveness of interventions in ITU and their core components to help improve family members’ satisfaction with care and their psychological health and well‐being. Future research might want to include family in the design of interventions, provide details of the implementation process and have clearly identified outcomes.

## 5 IMPLICATIONS FOR PRACTICE

- Family members’ need for information and assurance is perceived as being the most important need when their relative is admitted to the ICU. One major clinical implication of these results is that healthcare staff's ability to meet or satisfy these needs is not always achieved.
- Family members of patients who are admitted to ICU experience increased psychological burden, yet few studies were found on the effectiveness of interventions to improve their health and well‐being.
- Regular structured family meetings using targeted written and oral information are suggested to ensure families receive the informational support required. More research is needed in this area to add to the evidence base on the effectiveness of interventions to support family members in ICU

## 6 CONCLUSION

In conclusion, this scoping review identified four key themes that emerged from the literature. A key finding from this review is that studies of family need have received most attention and consistently identified the need for more information and reassurance. However, families’ perceived needs were not always met by healthcare staff and this had a negative impact on family satisfaction and their psychological health and well‐being. Whilst there is some evidence that interventions based on the provision of appropriate written and oral information in ICU can effectively reduce anxiety and improve satisfaction, more empirical research is needed in this area.

## CONFLICT OF INTEREST

No conflict of interest.
